# Community knowledge, attitude and practice about malaria in a low endemic setting of Shewa Robit Town, northeastern Ethiopia

**DOI:** 10.1186/1471-2458-13-312

**Published:** 2013-04-08

**Authors:** Andargie Abate, Abraham Degarege, Berhanu Erko

**Affiliations:** 1 Somali Regional State Health Office, P.O. Box 238, Jijga, Ethiopia; 2 Aklilu Lemma Institute of Pathobiology, Addis Ababa University, P.O. Box 1176, Addis Ababa, Ethiopia

**Keywords:** Malaria prevalence, Knowledge, Attitude, Practice, Shewa Robit, Ethiopia

## Abstract

**Background:**

Since malaria is one of the foremost public health problems in Ethiopia, assessment of situation of the disease, and communities’ knowledge and perceptions about malaria is necesary to institute appropriate preventive and control measures. Thus, the aim of this study was to assess malaria prevalence and knowledge, attitude and practice (KAP) about the disease among ShewaRobit Town community, northeastern Ethiopia.

**Methods:**

A community-based cross-sectional study was conducted in Shewa Robit Town from October to November 2011. A multi-stage random sampling technique was used to select the study participants. A total of 425 individuals were examined for malaria using thin and thick Giemsa stained blood film, and 284 of the participants were interviewed to assess their KAP about malaria. Logistic regression analysis was used to assess predictor factors for malaria prevalence.

**Results:**

All respondents had ever heard of malaria. Most of the respondents (85.2%) attributed the cause of malaria to mosquito bite. However, some of the respondents (>20%) mentioned lack of personal hygiene, exposure to cold weather, hunger, chewing maize stalk, body contact with malaria patient and flies as the causes of malaria. Sleeping under mosquito nets, draining stagnant water and indoor residual spraying were the most frequently mentioned malaria preventive measures perceived and practiced by the respondents. Among 425 individuals examined for malaria, only 2.8% were positive for *Plasmodium* parasites. Living in houses made of wall without hole, sprayed with insecticide within the last 12 hours and located at a distance of greater than 500 meters from potential mosquito breeding sites as well as knowing and using of mosquito net were significant predictors of low malaria prevalence among the study participants.

**Conclusions:**

A high level of knowledge about the cause, transmission and preventive methods of malaria was detected among the community in Shewa Robit Town. However, a considerable proportion had misconception about the cause and transmission of malaria suggesting the necessity of health education to raise the community’s awareness about the disease.

## Background

Malaria remains a major global public health and development challenge. It caused 216 million cases and 655,000 deaths worldwide in 2010, of which 81% of the cases and 91% of the deaths were from sub-Saharan Africa [1]. In Ethiopia, malaria also remains one of the most public health problems despite considerable effort made to control it [2-4]. Approximately 75% of the land mass where 68% of the total population lives is malarious [2]. The Federal democratic Republic of Ethiopia Ministry of Health estimated that there are more than 5 million clinical cases and thousands of deaths due to malaria each year [2]. However, the epidemiological pattern of the disease varies from place to place and even from time to time [5-7]. About 1 million confirmed clinical cases and 1,581 deaths were officially reported due to malaria in 2010 [1].

Due to availability of favorable conditions for the vector to develop and multiply, malaria tends to predominantly occur in rural areas [5,8,9]. However, studies documented increased malaria transmission in urban areas [10-13]. This could be associated with the rapid growth of cities coinciding with lack of proper sanitation, poor housing and poor drainage of surface water that facilitate human-mosquito interaction and subsequent malaria transmission [10-13].

Additionally, weak health services, increased migration of people from malarious rural areas to urban areas, limited tradition of indoor residual insecticide spraying (IRS) and bed net use, increased number of man-made mosquito breeding sites, and unplanned irrigation schemes and water collection reservoirs my hasten the spread of the disease in Ethiopian urban settings [5,13]. In view of the increasing urbanization in the country, there is a need to assess the risk of plasmodial infection. This provides baseline information to integrate malaria control activities with urban planning. This study assessed malaria prevalence and community KAP about the disease in Shewa Robit Town, northeastern Ethiopia.

## Methods

### Study area and population

A community-based cross-sectional study was conducted in Shewa Robit Town, northeastern Ethiopia, from October to November 2011. The town is located at 225 Km northeast of Addis Ababa, in the Amhara Regional State at an elevation of about 1,280 meters above sea level. The town lies at a longitude and latitude of 10°06^′^N39°59^′^E and10.1°N39.983°E, respectively. It is divided into 9 *kebeles* (lowest administrative unit) and several villages (sub units of *kebeles*). The town has a total population of 42,208 and 10,048 households, with the average of 4.5 family size. There are 10 governmental health centers, eleven private clinics and pharmacies. Malaria is one of the top ten diseases in the town and reported throughout the year [14].

Individuals eligible for the parasitological survey were all family members living in the 4 *kebeles* of Shewa Robit Town. However, only family members older than 18 years, who gave blood samples for malaria diagnosis, and volunteered for interview were included in the KAP survey about malaria. Relatives who joined familly members during the parasitological survey and mentally sick people were excluded from the KAP study. Family members who were not present at home during the study period and children who were younger than 18 years were also excluded from the KAP study.

### Sample size and sampling procedure

The sample size for the study was calculated using the formula (n = (zα/2)^2^ p (1-p)/ d^2^) for estimating a single population proportion at 95% confidence interval (CI) (Zα/2 = 1.96), 5% margin of error, design effect of 2, 15% non-response rates [15]. Based on a report of 14% prevalence of plasmodial infection among febrile individuals visiting a health center in the study area for medical attention [14], and an average family size of 4.5, a total of 426 individuals were selected for the parasitological survey from 94 households. The sample size for parasitological survey was calculated as n = [(1.96)^2^ (0.14) (0.86)/(0.05)^2^] = 185; total n = (1,85 × 2) + (1,85 × 2 × 0.15) = 370 + 56 = 4,26; and 426/4.5 = 95. Assuming an average of three individuals older than 18 years from each household, a total of 284 individuals were randomly selected from the 426 individuals for the KAP study.

A multi-stage cluster random sampling technique with *kebeles* as the first-stage unit, villages as the second, and households as the third stage was used to select the representative sample size. Four *kebeles* (clusters) were selected using a lottery method from the nine *kebeles* by the investigator. In each selected *kebele*, two villages (total 8) were randomly selected and households were selected from each study village in the respective *kebeles*. A total of 95 households were selected using probability proportion to size of households in the selected villages. The shared households for each village were divided by the total number of households in a given village to determine a sampling interval for selecting households. Accordingly, every 15^th^ households was selected using systematic random sampling technique.

### Data collection

A standard structured questionnaire was designed to collect information regarding socio-demographics and KAP of the study participants about malaria. The questionnaire was first developed in English and translated into Amharic (the local language), and then pre-tested in non-selected *kebeles* via pilot study for assessing content validity, appropriateness, and question comprehensibility. Then, the questionnaire was revised when necessary. Two nurses from the health center in the study area were selected to collect data. Training was given to the data collectors for two days on how to conduct the interview, content of the questionnaire, data quality, and ways to approach respondents. The first author checked the questionnaires for completeness every day. Incomplete questionnaires were returned to data collectors for correction by revisiting the households. Five percent of the interviewed households were randomly selected and re-interviewed by the first author.

*Plasmodium* infection was checked using finger prick thick and thin blood smears. Thin films were fixed with 100% methanol and both thin and thick films were stained with 3% Giemsa at health facility following a standard protocol [16]. Thick films were examined using high power magnification (100x) for the presence of *Plasmodium* parasites. When positive, thin film was examined for species identification. All positive slides and a random sample of 10% of the negative slides were re-examined by another experienced microscopist who was blinded to the diagnosis of the first reader. No discrepancy was identified between the readers.

### Data analysis

Data were double entered and cross-checked using Epi-data version 3.1, and analyzed using STATA version 11. Malaria prevalence was determined by dividing the number of *Plasmodium*-infected individuals by the total number of individuals examined for *Plasmodium* infection. Frequency distribution tables were used to quantify knowledge of respondents related to symptoms, causes, transmission, prevention and control measures of malaria. Fisher’s exact test was used to evaluate associations between malaria prevalence and KAP of respondents related to symptoms, causes, transmission, prevention and control measures of malaria. Multivariate logistic regression analysis was used to quantify the effect of different risk factors including KAP on malaria prevalence. Because of the nature of the outcome variable we assumed that there will be higher interclass correlation within *kebeles* hence, in logistic regression analysis we handled *kebele* as a clustering variable and we used robust standard error instead of maximum likely hood estimate. 95% confidence intervals were calculated for odds ratio. Values were considered statistically significant when *p* < 0.05.

### Ethical consideration

The study obtained ethical clearance from the Institutional Review Board (IRB) of Aklilu Lemma Institute of Pathobiology (ALIPB), Addis Ababa University. Supportive letter was obtained from the district health office before data collection, and written informed consent was obtained from voluntary participants and parents or guardians for children during data collection. Individuals who were found positive for *Plasmodium* parasite were treated with anti-malarial drugs as per the national guidelines.

## Results

### Socio-demographic characteristics

A total of 425 individuals provided blood samples for malaria diagnosis, of whom 284 (66.8%) were interviewed for their KAP about the disease (Table 1). Most of the interviewees were females (61.3%), married (60.6%), illiterate (50.0%), Amhara by ethnicity (89.1%) and aged 18 to 24 years (38.4%). Nearly 36% of respondents were farmers and 33.4% were students, daily laborers and house wives.

**Table 1:** Socio-demographic characteristics of the study participants, Shewa Robit Town, northeastern Ethiopia, 2011

**Variables**	**Category**	**Number of interviewees (%)**	**Number of examined for malaria (%)**
Sex	Male	110(38.7)	172(40.5)
	Female	174(61.3)	253(59.5)
Age	<5	NA	42(9.9)
	5-17	NA	99(23.3)
	18-24	109(38.4)	109(25.6)
	25-44	109(38.4)	109(25.6)
	≥45	66(23.2)	66(15.5)
Marital Status	Single	83(29.2)	NA
	Married	172(60.6)	NA
	Divorced	21(7.4)	NA
	Widowed	8(2.8)	NA
Education	Illiterate	142(50.0)	NA
	Elementary and Junior	87(30.6)	NA
	Secondary and above	55(19.4)	NA
Occupation	Farmer	103(36.3)	NA
	Merchant	52(18.3)	NA
	Government employee	34(12.0)	NA
	Others	95(33.4)	NA
Ethnic group	Amhara	253(89.1)	375(88.2)
	Argoba	29(10.2)	48(11.3)
	Others	2(0.7)	2(0.5)

Note: NA = Not applicable.

### Knowledge, attitude and practice (KAP) about malaria

Malaria is known as “*webba*” in Amharic which is most commonly used term in the study area. All respondents (284) had ever heard of malaria and 93.0% of them believed that malaria was one of the major health problems of the community. Fever and chills were most frequently mentioned symptoms reported by 94.4% and 93.3% of the respondents, respectively. Most of the respondents (85.2%) attributed the cause of malaria to mosquito bite. However, more than 20% of the respondents associated the cause of malaria with chewing maize stalk, hunger, lack of personal hygiene and exposure to cold weather. Mosquitoes are mainly believed to bite humans at night during sleeping time (83.8%), breed in stagnant water (91.6%) and rest at the edges of streams during daytime (51.4%) (Table 2).

**Table 2:** Knowledge and attitude of respondents related to symptoms and causes of malaria, and mosquito behaviors, Shewa Robit Town, northeastern Ethiopia, 2011

**Variables**	**Category**	**Frequency (%)**
Cause of malaria mentioned	Mosquito bite	242(85.2)
	Lack of personal hygiene	66(23.2)
	Cold weather	39(13.7)
	Hunger	94(33.1)
	Chewing maize stalk	96(33.8)
Signs/symptoms of malaria	Fever	268(94.4)
	Chills	265(93.3)
	Headache	240(84.5)
	Back pain/Joint pain	197(69.4)
	Nausea/Vomiting	189(66.6
	Loss of appetite	173(60.9)
	Thirsty	196(69.0)
	Don’t know	2 (0.7)
When mosquitoes bite mostly	Day	5(1.8)
	Night	238(83.8)
	Any time	26(9.1)
	Don’t know	15(5.3)
Common breeding sites	Stagnant water	260(91.6)
	Running water	10(3.5)
	Waste materials	103(36.3)
	Vegetation	48(16.9)
	Houses	21(7.4)
	Don’t know	6(2.1)
Common resting sites	Dark place inside houses	91(32.0)
	At edges of streams	146(51.4)
	Dirty areas	104(36.6)
	Don’t know	24(8.4)

Note: Percentages do not add up to 100 because of multiple responses.

About 48% of the respondents mentioned that malaria could be transmitted from person to person of whom 95.6% linked the transmission with mosquito bite. All respondents replied that malaria is a preventable disease. Sleeping under mosquito net (93.7%), draining stagnant water (84.2%) and IRS (78.9%) were the most frequently mentioned malaria preventive methods by the study participants. Most respondents believed that mosquito nets protect from mosquito bite (Table 3). Twenty percent of the respondents replied that using mosquito net is not comfortable as it results in hot sleeping and/or bad smell.

**Table 3:** Knowledge and attitude of respondents regarding transmission and preventive methods of malaria, Shewa Robit Town, northeastern Ethiopia, 2011

**Variables**		**Frequency (%)**
Mode of transmission	Through mosquito bite	130(95.6)
	Through bodily contact with patients	15 (11.0)
	Via respiratory route	4(2.9)
	By flies	2(1.5)
Preventive methods	Take tablets	151(53.2)
	House spray with insecticide	224(78.9)
	Drain stagnant water	239(84.2)
	Clear the vegetation	130(45.8)
	Use of mosquito net	266(93.7)
	Fumigation	149(52.5)
	Closing windows and doors	166(58.4)
Advantage of mosquito nets	Protect from mosquito bite	248(87.3)
	Avoid getting malaria	201(70.8)
	Protect from other insects	148(52.1)
	Sleep better	80 (28.2)
	Warmer	27 (9.5)

Note: Percentages do not add up to 100 because of multiple responses.

Out of the total respondents, 70.4% reported that their houses were sprayed with DDT in the last 12 months, and about 30% mentioned that their houses were not sprayed within the last 12 months. Among those who had regular IRS, the respondents mentioned that IRS can kill mosquito (75.0%) and other insects (23.5%), thereby protecting from malaria (66.5%). The majority of the interviewees (96.5%) responded that they prefer to get health service from public health facilities than from private ones. Most of the respondents practiced use of mosquito nets (87.7%) and house spray with insecticide (59.5%) to prevent and control malaria (Table 4).

**Table 4:** Practices of respondents towards malaria prevention and control, Shewa Robit Town, northeastern Ethiopia, 2011

**Variables**	**Frequency (%)**
Take tablets	66(23.2)
House spray with insecticide	169(59.5)
Drain stagnant water	133(46.8)
Clear the vegetation	41(14.4)
Use of mosquito net	246(86.6)
Fumigation	101(35.6)
Closing windows and doors	116(40.8)
Don’t use	4(1.4)

Note: Percentages do not add up to 100 because of multiple responses.

### Prevalence of malaria

Out of the total 425 participants examined for *Plasmodium* parasites, only 12 (2.8%, 95% CI: 1.2-4.4) had the parasite in their blood. *P. vivax* accounted for 11(91.7%) of the cases and *P. falciparum* for the remaining one case (8.3%).

The odds of *Plasmodium* infection was significantly lower among communities living in houses located at a distance of greater than 500 meters from mosquito breeding sites compared to those who live in houses placed ≤500 meters from mosquito breeding sites (adjusted OR = 0.06; 95% CI: 0.01-0.22). Similarly, the chance of *Plasmodium* infection was significantly lower among communities living in houses without holes in their walls compared to those who live in houses with holes in their walls (adjusted OR =0.08; 95% CI: 0.01-0.58). The odds of malaria was also significantly lower in individuals who use ITNs than those who didn’t use (adjusted OR = 0.17, 95% CI: 0.06, 0.50) The odds of *Plasmodium* infection was significantly higher in individuals whose house was not sprayed with insecticide compared to those whose house was sprayed with insecticide within the last 12 months (adjusted OR =2.31, 95%CI: 1.98-2.69). However, prevalence of malaria was comparable among communities living in houses with window screening versus in communities living in houses without window screening, and individuals of age group < 5 years versus those aged 5 to 14 years and ≥15 years (Table 5).

**Table 5:** Malaria prevalence stratified by different risk factors among community members, Shewa Robit Town, northeastern Ethiopia, 2011

**Variables**		**Number**		**AOR(95%CI)**
		**Examined**	**Positive (%)**	
Age	<5	42	4(9.5)	1.00
	5-14	79	1(1.3)	0.12(0.01, 1.50)
	≥15	304	7(2.3)	0.79(0.04,14.95)
Sex	Male	172	8(4.6)	1.00
	Female	253	4(1.6)	0.48 (0.07, 3.55)
Distance of breeding site	≤500 ms	123	9(7.3)	1.00
	>500 ms	157	2(1.3)	0.06(0.01,0.22)**
DDT spray	Yes	297	6(2.0)	1.00
	No	128	6(4.7)	2.31 (1.98,2.69)**
Presence of ITN	No	37	3(8.1)	1.00
	Yes	388	9(2.3)	0.17 (0.06, 0.50)*
Screened windows	Yes	242	3 (1.2)	1.00
	No	103	7(6.8)	2.16 (0.89,5.24)
Presence of hole in the wall	Yes	153	11(7.2)	1.00
	No	272	1(0.4)	0.08(0.01, 0.58)*

** = significant at <0.001 level; * = significant at <0.05 level; AOR = adjusted odds ratio.

Adjusted OR (adjusted odds ratio from multivariable logistic regression model) = when the effect of one factor on malaria prevalence is evaluated the analysis was adjusted for other remaining factors listed in the table. In addition, the analysis was adjusted for the clustering effect of *kebeles* using robust standard error.

Prevalence of malaria was significantly lower among the interviewees who responded that they use ITN to prevents malaria compared to those who were not aware that ITN can prevent malaria (Fisher exact = 0.01). Prevalence of malaria was also significantly lower among interviewees who actually used ITN compared to those who did not use ITN (Fisher exact = 0.03). However, prevalence of malaria was similar among respondents who used to drain stagnant water, clear vegetation and spray their houses with insecticides compared to those who did not practice these measures (Table 6). Prevalence of the disease was also similar among interviewees who mentioned draining stagnant water, clearing vegetation and house spraying with insecticides as preventive measures for malaria compared to those who were not aware of these practices (Table 6).

**Table 6:** Association of selected KAP related variables with malaria prevalence, Shewa Robit Town, northeastern Ethiopia, 2011

**Variables**	**Number of examined (N = 284)**		**Positive (%)**	**Fisher’s exact**
Mosquito bite causes malaria	Yes	242	4(1.6)	0.22
	No	42	2(4.8)	
Malaria is transmissible disease	Yes	136	1(0.7)	0.22
	No	148	5(3.4)	
**Preventive measures**				
House spray with insecticide	Yes	224	5(2.2)	1.00
	No	60	1(1.7)	
Drain stagnant water	Yes	239	5(2.1)	1.00
	No	45	1(2.2)	
Clear the vegetation	Yes	130	3(2.3)	1.00
	No	154	3(2.0)	
Use of mosquito net	Yes	266	3(1.1)	0.01
	No	18	3(16.7)	
**Practice**				
House spray with insecticide	Yes	169	2(1.2)	0.23
	No	115	4(3.5)	
Drain stagnant water	Yes	133	3(2.3)	1.00
	No	151	3(2.0)	
Clear the vegetation	Yes	41	2(4.9)	0.21
	No	243	4(1.7)	
Use of mosquito net	Yes	246	3(1.2)	0.03
	No	38	3(7.9)	

## Discussion

Community-based cross-sectional study on malaria prevalence and communities’ knowledge, attitude and practice towards the cause, transmission, prevention and control measures of the disease was undertaken in Shewa Robit Town, northeastern Ethiopia. The study revealed lower malaria prevalence (2.8%) compared to reports from Jimma Town, southwestern Ethiopia (5.2%) [13] and Kenya (18.0%) [17]. Environmental variation, type of study population and level of endemicity may explain this difference [7,18,19]. Our study participants were all age groups, but those of Kenya were pregnant women who are more susceptible to malaria attacks [17].

The predominant *Plasmodium* species detected among the current study participants was *P. vivax*. Only one individual was positive for *P. falciparum*. This is in agreement with the previous report from Jimma Town [13] possibly due to the dominance of *P. vivax* over *P. falciparum* in recent years [20].

IRS with DDT, use of mosquito nets, lack of holes in living houses and good knowledge and practice of participants in preventing malaria were significantly associated with a lower prevalence of malaria which agrees with reports from other parts Ethiopia [21], Eritrea [22] and Kenya [17]. Similarly, decreased malaria prevalence was observed among community members living far from breeding sites which agrees with previous report from Ethiopia [21] and western Kenya [23]. Woyesa et al. [10] also observed an inverse relationship between mosquito density in village and the distance of settlement from the river suggesting that mosquitoes tended not to fly far from breeding sites for blood meals.

The questionnaire survey results showed that all respondents had ever heard of malaria and more than 90% of them believed that malaria was one of the most important health problems of the community affecting both sex and all age groups, which is consistent with previous reports [24,25]. Most of the respondents were also familiar with at least one of the classical symptoms of malaria which is expected for a population in endemic areas where people are aware of the clinical manifestations of the disease [26,27]. However, the lower prevalence of malaria (2.8%) observed among the study participants could be due to different malaria preventive and control measures being implemented in the area. Currently, the government is undertaking control measures, including distribution of ITN and residual spraying of houses with insecticides in malarious regions of Ethiopia. This may contribute to the reduced malaria prevalence observed among communities in the current study area.

The majority of the respondents mentioned that mosquito bite was a possible cause of malaria, and transmission occurs from person-to-person through mosquito bite. This awareness is higher than the level reported in other parts of Ethiopia [24,26,28]. The difference might be due to the presence of accessible house-to-house health extension services, which focuses on information, education and communication (IEC)/behavioral change communication in the current study area. In Ethiopia, regular practice of creating awareness in communities about health issues through health extension workers has started in recent years.

Although majority of the respondents associated the cause of malaria with mosquito bite, more than 20% of respondents mentioned lack of personal hygiene, exposure to cold weather, hunger, chewing maize stalk, body contact with malaria patient, flies and respiratory route as the causes of malaria. Such misunderstandings have also been reported by other studies [24,28-31]. Although the belief of the community that exposure to cold weather, hunger and chewing maize stalk as direct causes of malaria seem incorrect, the idea may stem from the occurrence of other related factors with stated condition which could be risk factors for malaria. For example, cold and cloudy weather could be related to the presence of mosquito breeding sites. In addition, maize pollen is released following rainy season which can be used as food source for larval stage enhancing their development and survival. Thus, malaria transmission could be accelerated in cold weather during winter seasons where maize is common [32]. Hunger could also lead to poor nutritional status which could make individuals more susceptible to malaria [33,34]. Despite these facts, perception of these by the study participants as direct causes of malaria may influence the actual prevention mechanism they may choose. Thus, this should be corrected with appropriate health education which could change their behavioral. Studies have reported better understanding of the causes of malaria in communities who had better awareness about the issue through health education [30,35].

More than half of our study participants mentioned mosquitoes’ habit of biting during sleeping time, breeding in stagnant water as well as resting at edges of streams. However, in rural parts of central Ethiopia, night biting habit of mosquitoes and breeding in stagnant water were reported by 42.6% and 36.2% of the respondents, respectively [24]. This correct perception among respondents of the present study is encouraging to take appropriate preventive measures and proper use of mosquito nets.

The most common sources of treatment mentioned by respondents in this study were health facilities. This is consistent with other observations in other African countries and India where health facility services were preferred most frequently when malaria is suspected [19,25,36]. Nevertheless, some of the respondents claimed to have used traditional medicines which is similar with previous reports from Sudan [19] and Bangladesh [37].

In this study, all respondents believed that malaria is preventable disease which is in contrast to the finding of a study in other areas of Ethiopia (85.7%) [26]. Use of mosquito nets, filling and draining mosquito breeding sites (stagnant and surface water) and house spray with insecticide were the three main types of malaria preventive measures frequently reported by the current study participants. This agrees with previous observations from other parts of Ethiopia, Tanzania and Bangladish [28,31,37]. A great number of respondents (94.7%) knew that ITNs prevent malaria, and hence made use of them to prevent the disease. More than half of respondents also mentioned IRS as a preventive measure and that they get their houses sprayed during spraying campaign. These could be some of the reasons for the low malaria prevalence detected among the present study participants However, about 45% the respondents usually re-plaster their houses after spraying with insecticide. This suggests the necessity of health education to raise community awareness about proper uses of preventive measures. The present study participants also practiced environmental management (filling and drainage of mosquito breeding sites) activities as preventive measures for malaria.

The limitations of this study include failure to use qualitative method and direct observation of ITNs usage. Immunological, genetic, nutritional and health status (e.g. occurrence of co-infections) of the study participants, which could affect status of *Plasmodium* infections were not considered. In addition, the number of malaria cases was too small for making firm conclusion regarding predictors of malaria prevalence.

## Conclusions

In conclusion, it is evident that the community’s overall awareness about the cause, symptoms and preventive measures of malaria was found to be high, and prevalence of malaria was found to be low in Shewa Robit Town. However, knowledge gaps about the cause and transmission of malaria were also observed among the residents in the area. Thus, appropriate health education should be implemented to correct misconceptions about the cause and transmission of malaria, and attention should be focused on the residents near to breeding sites.

## Competing interests

The authors declare that they have no competing interests.

## Authors’ contribution

AA, AD & BE: Conceived and designed the study. AA: collected and analyzed the data. AA, AD & BE drafted the manuscript. Finally all authors commented and approved the final manuscript.

## Pre-publication history

The pre-publication history for this paper can be accessed here:

http://www.biomedcentral.com/1471-2458/13/312/prepub
